# Progress in *LRRK2*-Associated Parkinson’s Disease Animal Models

**DOI:** 10.3389/fnins.2020.00674

**Published:** 2020-07-15

**Authors:** Steven P. Seegobin, George R. Heaton, Dongxiao Liang, Insup Choi, Marian Blanca Ramirez, Beisha Tang, Zhenyu Yue

**Affiliations:** ^1^Department of Neurology, Icahn School of Medicine at Mount Sinai, New York, NY, United States; ^2^Department of Neuroscience, Icahn School of Medicine at Mount Sinai, New York, NY, United States; ^3^Friedman Brain Institute, Icahn School of Medicine at Mount Sinai, New York, NY, United States; ^4^Department of Neurology, Xiangya Hospital, Central South University, Hunan, China; ^5^National Clinical Research Center for Geriatric Disorders, Xiangya Hospital, Central South University, Hunan, China

**Keywords:** LRRK2, models, rodent, *Drosophila*, *C. elegans*, zebrafish, phenotypes

## Abstract

Mutations in the leucine-rich repeat kinase 2 (*LRRK2*) gene are the most frequent cause of familial Parkinson’s disease (PD). Several genetic manipulations of the *LRRK2* gene have been developed in animal models such as rodents, *Drosophila*, *Caenorhabditis elegans*, and zebrafish. These models can help us further understand the biological function and derive potential pathological mechanisms for LRRK2. Here we discuss common phenotypic themes found in *LRRK2*-associated PD animal models, highlight several issues that should be addressed in future models, and discuss emerging areas to guide their future development.

## Introduction

Parkinson’s disease (PD) is a common, age-dependent, neurodegenerative disease with a lifetime risk of approximately 1.5% (Lees et al., 2009). The defining diagnostic features of PD include bradykinesia, resting tremor, rigidity, cognitive impairment, and psychiatric dysfunction (Goetz et al., 2008). Neuropathologically, PD is characterized by degeneration of dopaminergic (DA) neurons in the substania nigra pars compacta (SNpc) and the presence of Lewy Bodies (LBs) throughout the central nervous system (CNS) (Gelb et al., 1999; Dickson et al., 2009). Extensive human genetic studies have led to the discovery of several PD-causing genes, including leucine-rich repeat kinase 2 (*LRRK2*) (Nalls et al., 2019). PD-causing mutations in *LRRK2* occur in up to 41% of select patient populations and, as such, they represent the greatest known cause of heritable PD (Khan et al., 2005; Lesage and Brice, 2009). Mutations in *LRRK2* are also found in sporadic PD, occurring at a rate of 1–3% (Gilks et al., 2005; Lesage et al., 2007; Paisán-Ruíz et al., 2008). Importantly, the clinical presentation of Parkinsonism in *LRRK2* mutation carriers has been described as indistinguishable from sporadic PD patients (Adams et al., 2005; Kay et al., 2006). Given its importance in both sporadic and familial PD, understanding LRRK2 biology will assist in elucidation of common mechanisms of disease pathogenesis (for a more thorough review, see Kluss et al., 2019).

The *LRRK2* gene encodes a large, 2,527-amino acid, 286-kDa, multi-domain protein belonging to the ROCO family (Zimprich et al., 2004). All ROCO proteins are characterized by a GTPase Ras-like G domain (Roc), followed in tandem by a C-terminal of Roc domain (COR) (Bosgraaf and Van Haastert, 2003; Marín, 2006). LRRK2 also contains a serine-threonine kinase domain, capable of phosphorylating both itself and a small group of substrates (West et al., 2005; Sheng et al., 2012; Steger et al., 2016). To date, most of the pathogenic mutations are clustered within the Roc, COR, or kinase domains and are found to alter LRRK2’s biochemical activity (Chen and Wu, 2018). Although LRRK2 activity has been linked to a diverse range of cellular processes (reviewed by Berwick et al., 2019), a large body of work suggests LRRK2 plays a key role in membrane trafficking along the endo-lysosomal pathway. These functions include synaptic vesicle endocytosis, degradation, and recycling of *trans*-membrane receptors, anterograde trafficking of receptors from the *trans*-golgi network to lysosomes, and retromer-mediated transmembrane recycling. LRRK2-dependent regulation of these cellular processes may be associated with LRRK2’s ability to bind and phosphorylate a cluster of Rab GTPases (Shin et al., 2008; Piccoli et al., 2011; MacLeod et al., 2013; Gómez-Suaga et al., 2014; Schreij et al., 2014; Pan et al., 2017; Steger et al., 2017; Pellegrini et al., 2018; Rivero-Ríos et al., 2019; Sheehan and Yue, 2019).

A diverse range of animal models have been developed which overexpress, knock-out, knock-down, or knock-in mutated and wildtype forms of the *LRRK2* gene, for the characterization of LRRK2 biological and pathophysiological functions. In this review, we discuss common phenotypic themes found in *LRRK2*-associated rodent, *Drosophila*, *Caenorhabditis elegans*, and zebrafish animal models and highlight several new avenues toward development of future *LRRK2* animal models.

## Rodent *LRRK2* Models

Rodent models have been widely used in the study of LRRK2 biology due to their genetic and neuroanatomical similarities to humans. Both mice and rats possess a mammalian homolog of *LRRK2* which shares approximately 86–88% sequence identity to human LRRK2. Importantly, all residues affected by pathogenic mutations in humans are conserved in rodent *LRRK2* (Langston et al., 2016). Rodents also possess a LRRK1 homolog, which shares ankyrin repeats (ANK), leucine-rich repeats (LRR), and Roc, COR, and kinase domains with LRRK2, but may also contain a WD40 domain that is still contested in the literature (Biskup et al., 2007; Civiero et al., 2012; Sejwal et al., 2017; Xing et al., 2017). The dopaminergic neuroanatomical pathways of rodents and humans are also highly similar, leading to the development of an array of sensitive behavioral tests in rodents that may correlate to dopamine loss in human PD (Meredith and Kang, 2006; Redgrave et al., 2010). Therefore, rodents represent an ideal candidate for genetic manipulations to investigate LRRK2 biology toward investigation of PD pathogenesis.

### Rodent *LRRK2* KO Models

*LRRK2* knock-out (KO) models have been chiefly employed to investigate the physiological function of endogenous LRRK2 (Tables 1, 2). An emerging theme of *LRRK2* KO rodent models is that the resulting phenotypes do not mimic *LRRK2*-assocated PD. Rodents born without *LRRK2* exhibit no loss of dopaminergic (DA) neurons, demonstrate mild to no behavioral or locomotor defects, have limited neuropathology, and have unchanged dopamine synthesis (as measured by DOPAC and HVA) (Andres-Mateos et al., 2009; Lin et al., 2009; Tong et al., 2010b; Herzig et al., 2011; Hinkle et al., 2012; Daher et al., 2014).

**TABLE 1:** Mouse *LRRK2* KO models.

Mouse *LRRK2* KO models	Author(s), year(s)	Model	Background (strain)	DA neuronal loss	Locomotor/behavioral changes	Other notes
1	Giaime et al., 2017	Double KO of *LRRK1* and *LRRK2* KO mouse [mouse *LRRK2* (−/−)/mouse *LRRK1* (−/−)].	Mouse C57BL/6J and 129 hybrid.	Loss at 14–15 months in SNpc and LC. Loss of medium spiny neurons in striatum.	Not assessed.	Presence of synuclein pathology. Increased p62 and LC3 in brain at 15 months. Increased electron dense vacuoles in SNpc at 10 months.
2	Dächsel et al., 2010; Hinkle et al., 2012; Volta et al., 2015	*LRRK2* KO by removal of exon 41 of *LRRK2* [mouse *LRRK2* (−/−)].	C57BL/6J [(also contains C57BL/6N – (Yue et al., 2015)]	No loss in SN at 18 months (Hinkle).	Increased thigmotaxis (open field), decreased center exploration time (open field) and decreased object approaches (novel object test) at 7 and 16 months. Increased latency to fall (rotarod) at 7 months (Hinkle).	No synuclein or tau pathology in kidney or brain at 3, 12, and 18 months. Progressive kidney degeneration seen at 3 months with increased lipofuscin or lysosomes. Increased autophagy markers at 20 months (p62 at 12 months) (Hinkle). Increased neurite outgrowth in hippocampal and midbrain neuron cultures (Dächsel).
3	Herzig et al., 2011	*LRRK2* KO using cre-recombinase deletion [mouse *LRRK2* (−/−)].	C57BL/6J or BALB/c.	Not assessed.	Not assessed.	Darkened kidney. Increased weight and vacuoles at 5 months. Increase of secondary lysosomes in the kidney and lung cells at 1.5 months. Increased diastolic blood pressure.
4	Tong et al., 2010a, 2012	*LRRK2* KO by deletion of promoter and exon 1 [mouse *LRRK2* (−/−)].	C57BL/6J and 129 hybrid.	No loss up to 24 months.	Not assessed.	Smaller size, increased synuclein aggregation, increased p62, increased LC3-I, decreased LC3-II, and increased apoptosis in the kidneys at 20 months (Tong et al., 2010a). Increased weight (kidney/body weight) and size of kidney at 1, 4, and 7 months. Decreased HMW α-synuclein, decreased LC3-I, and decreased p62 at 7 months. Increased kidney injury molecule-1 and cathepsins (Tong et al., 2012).
5	Andres-Mateos et al., 2009	*LRRK2* KO by partial deletion of exon 39, complete deletion of exon 40 and insertion of premature stop codon [mouse *LRRK2* (−/−)].	C57BL/6.	No loss in SN up to 18–22 months.	Not assessed.	Lack of sensitivity to MPTP.
6	Lin et al., 2009; Parisiadou et al., 2009	*LRRK2* KO by deletion of exon 2 resulting in premature stop codon in exon 3 [mouse *LRRK2* (−/−)].	C57BL/6J.	No obvious degeneration (not directly assessed).	No changes in open field and rotarod (Lin).	Protection against α-synuclein aggregation in the striatum (Lin).

**TABLE 2:** Rat *LRRK2* KO models.

Rat *LRRK2* KO models	Author(s), year(s)	Model	Background (strain)	DA neuronal loss	Locomotor/behavioral changes	Other notes
1	(Baptista et al., 2013; Ness et al., 2013; Daher et al., 2014; Miklavc et al., 2014; Arranz et al., 2015; Boddu et al., 2015) (MJFF Comparison Study – Rats) (Charles River-SAGE).	*LRRK2* KO by 10 bp deletion in exon 30 resulting in premature stop codon [rat *LRRK2* (−/−)].	Outbred Long-Evans.	No loss, age not indicated (Daher).	*LRRK2* KO rats show increased forelimb and hindlimb grip strength and decreased TH+ neurons in SN (MJFF Comparison study of rats - data not published caution required).	*LRRK2* KO rats are protective from α-synuclein injection (Daher). Increased body weight (Ness). *LRRK2* KO primary neurons have slowed clathrin-mediated endocytosis and decreased SVs *in vitro* (Arranz).

Although no clear neuronal phenotypes have emerged, several studies have suggested that LRRK2 may fulfill key functions in peripheral tissues. Most notably, *LRRK2* KO kidneys exhibit striking age-dependent changes in color and weight, along with ultrastructural abnormalities (Tong et al., 2010a, 2012; Herzig et al., 2011; Hinkle et al., 2012; Baptista et al., 2013). A darker coloration of *LRRK2* KO kidneys has been observed as early as 2–3 months, while increases in weight are reported as early as 1–5 months in a sex-specific manner (Herzig et al., 2011; Hinkle et al., 2012; Tong et al., 2012; Ness et al., 2013; Boddu et al., 2015). Curiously, one group has reported a decrease in the weight of *LRRK2* KO kidneys at 20–27 months in age (Tong et al., 2010b, 2012). Electron microscopy (EM) analysis of KO kidneys has further revealed an increase in the number and size of lysosomes in epithelial cells of the renal cortex, starting at 4 months of age (Herzig et al., 2011; Hinkle et al., 2012; Tong et al., 2012; Baptista et al., 2013). These studies also found increases in lipid-containing vesicles or droplets and lipofuscin (potentially contained within lysosomes). Alongside ultrastructural abnormalities, multiple groups have reported changes in autophagic markers, LC3, and p62 and lysosomal markers, LAMP-1 and Cathepsin D, consistent with alterations in the autophagy–lysosomal pathway (Tong et al., 2010a, 2012; Hinkle et al., 2012). Changes in homeostatic parameters regulated by the kidneys have also been reported in *LRRK2* KO animals, including increased diastolic blood pressure, decreased plasma and serum chloride, and decreased specific gravity of urine (Herzig et al., 2011; Baptista et al., 2013; Ness et al., 2013; Boddu et al., 2015; Fuji et al., 2015). Kidney dysfunction may be detected using the biomarker of kidney health known as lipocalin-2 (NGAL), which has also been observed to be reduced in plasma and urine (Ness et al., 2013; Fuji et al., 2015).

Several additional abnormalities have been reported in other peripheral organs in *LRRK2* KO animals. Lungs of *LRRK2* KO rats exhibit an increased number and size of type II alveolar cell lamellar bodies, a lysosome-derived secretory vesicle that stores surfactant (Herzig et al., 2011; Baptista et al., 2013; Miklavc et al., 2014). Indications of liver dysfunction such as increased AST, ALT, and cholesterol levels have also been reported (Ness et al., 2013; Baptista et al., 2013). To a lesser degree, abnormalities in the spleen, such as decreased size and changes in cellular composition, were also found (Ness et al., 2013).

A less-clear phenotype seen has been the disruption of α-synuclein homeostasis in *LRRK2* KO rodent models. While several studies have shown that *LRRK2* KO or kinase inhibition is protective against α-synuclein aggregation in the brain (Lin et al., 2009; Herzig et al., 2011; Daher et al., 2014; Bae et al., 2018), others have reported that *LRRK2* KO causes accumulation of α-synuclein in the kidneys (Tong et al., 2010a, 2012). These seemingly conflicting findings may indicate tissue or model specific differences in α-synuclein metabolism.

An exception to phenotypes seen in single *LRRK2* KO models has been a recently developed *LRRK1* and *LRRK2* double-KO mouse that shows α-synuclein pathology, disruption of the autophagy–lysosomal pathway, and DA neurodegeneration in the CNS (Giaime et al., 2017). While the result suggests the possibility that LRRK1 compensates for the loss of LRRK2 in the brain, it raises the question as to whether PD mutations of *LRRK2* could be implicated in the loss of LRRK1/LRRK2 functions. Future studies will have to further verify these findings and elucidate potential mechanisms.

### Rodent Transgenic *LRRK2* Models

Several rodent transgenic models have been generated to overexpress (OE) WT or pathogenic variants of *LRRK2*. These transgenic models have been developed using either insertions of *LRRK2* cDNA or bacterial artificial chromosomes (BACs) (Tables 3–5).

**TABLE 3:** Transgenic OE of *LRRK2* using cDNA in mouse models.

Transgenic overexpression cDNA Mouse Models	Author(s), year(s)	Model (species, gene, WT or mutant, tag)	Background (strain)	Type of expression system	DA neuronal loss	Locomotor/behavioral changes
1	Xiong et al., 2018	Human *LRRK2* G2019S – TAP.	C57BL/6 (backcrossed).	Tet-inducible OE of human *LRRK2* G2019S with human TH promoter (catecholaminergic cell specific).	Loss at 15 months in SNpc and 24 months in LC.	Decreased stride length and increased descending time on the pole test at 24 months. Normal rotarod and open field.
2	Xiong et al., 2018	Human *LRRK2* GS/Kinase Dead (G2019S + D1994A) – TAP.	C57BL/6 (backcrossed).	Tet-inducible OE of human *LRRK2* kinase dead with human TH promoter (catecholaminergic cell specific).	No loss in SNpc or LC up to 25 months.	No changes on rotarod, open-field, pole test, and gait analysis.
3	Weng et al., 2016	Human *LRRK2* R1441C – HA.	FVB/N.	OE of human *LRRK2* R1441C under CMVE/(PDGF)-β promoter (neuronal transgene specific).	Loss in SNpc at 16 months. No neuronal loss in striatum up to 16 months.	Decreased velocity, distance moved and rearings starting at 16 months.
4	Liu et al., 2015	Human *LRRK2* WT – HA.	C57BL/6J.	Tet-inducible OE of human *LRRK2* WT with PITX3 promoter (DA midbrain neuron specific).	No loss in SNpc or VTA up to 20 months.	No changes in open field, rotarod, and gait analysis up to 18 months.
5	Liu et al., 2015	Human *LRRK2* G2019S – HA.	C57BL/6J	Tet-inducible OE of human *LRRK2* G2019S LRRK2 with PITX3 promoter (DA midbrain neuron specific).	No loss in SNpc or VTA up to 20 months.	No changes in gait analysis, rotarod, fine movement and horizontal/vertical movement.
6	(Garcia-Miralles et al., 2015) [Note: (Herzig et al., 2012) developed a similar model]	Human *LRRK2* WT.	C57BL/6.	OE of human *LRRK2* WT with murine Thy1.2 promotor (neuronal transgene specific).	No loss in SNpc up to 12–13 months (limited expression in SNpc).	Not assessed.
7	(Garcia-Miralles et al., 2015) [Note: (Herzig et al., 2012) developed a similar model]	Human *LRRK2* G2019S.	C57BL/6.	OE of human *LRRK2* G2019S with murine Thy1.2 promoter (neuronal transgene specific).	No loss in SNpc up to 12–13 months (limited expression in SNpc).	Not assessed.
8	Tsika et al., 2014	Human *LRRK2* R1441C.	C57BL/6J.	Conditional (Cre-dependent) OE of human LRRK2 R1441C with murine ROSA26 promoter (crossed with DAT-Cre for central DAT-positive midbrain neuron expression).	No loss in SNpc up to 22 months.	No changes in open field, rotarod or olfactory sense at 19–20 months.
9	Maekawa et al., 2012	Human *LRRK2* I2020T – V5.	C57BL/6J (backcrossed).	OE of human *LRRK2* I2020T with CMV promoter (whole body expression).	No loss in SNc up to 18 months.	Increased slips on beam test (23 weeks), decreased latency to fall time (34 weeks), and increased rearings (22 weeks).
10	Chen et al., 2012; Weng et al., 2016	Human *LRRK2* WT – HA.	FVB/N.	OE of human *LRRK2* WT with CMVE/(PDGF)-β promoter (neuronal transgene specific).	No loss in SNpc. No neuronal loss in striatum, cerebral cortex, or hippocampus.	No changes in open field and pole test.
11	Chen et al., 2012; Chou et al., 2014	Human *LRRK2* G2019S – HA.	FVB/N.	OE of human *LRRK2* G2019S with CMVE/(PDGF)-β promoter (neuronal transgene specific).	Loss in SNpc starting at 12 months (50%). No neuronal loss seen in striatum, cerebral cortex, or hippocampus (Chen). No loss at 8–9 months in SNpc (Chou).	Decreased ambulatory movement, distance moved, and increased time on pole test at 12 months (Chen). Decreased ambulatory movement at 8–9 months (Chou).
12	Herzig et al., 2012	Human *LRRK2* WT.	C57BL/6	OE of human *LRRK2* WT with murine Thy1 promoter (neuronal transgene specific).	Not assessed (limited expression in SNpc).	Trend for increased rotarod and distance traveled, but not significant (data not shown).
13	Herzig et al., 2012	Human *LRRK2* G2019S.	C57BL/6.	OE of human *LRRK2* G2019S with murine Thy1 promoter (neuronal transgene specific).	No obvious pathology up to 19 months due to lack of expression in SN (data not shown).	Increased rotarod performance at 3–4 months and increased distance traveled in first 30 min at 7 months (males only). These effects waned later in age (data not shown for rotarod).
14	Ramonet et al., 2011	Human *LRRK2* WT.	C57BL/6J (backcrossed).	OE of human *LRRK2* WT with CMVE/human (PDGF)-β promoter (neuronal transgene specific).	Not assessed (limited expression in SNpc).	Not assessed.
15	Ramonet et al., 2011	Human *LRRK2* R1441C.	C57BL/6J (backcrossed).	OE of human *LRRK2* R1441C with CMVE/human (PDGF)-β promoter (neuronal transgene specific).	Not assessed (limited expression in SNpc).	Decreased horizontal and vertical activity measured by beam breaks at 15 months.
16	Ramonet et al., 2011; Lim et al., 2018	Human *LRRK2* G2019S.	C57BL/6J (backcrossed).	OE of human *LRRK2* G2019S with CMVE/human (PDGF)-β promoter (neuronal transgene specific).	Loss in SNpc at 19–20 months (17–18%). No loss in VTA at 19–21 months (Ramonet).	No changes in open field, beam test or acoustic startle response up to 15 months (Ramonet). Decreased latency to fall time on rotarod at 65–83 weeks and Increased anxiety/depression 43–52 weeks (Lim).
17	Lin et al., 2009	Human *LRRK2* WT – HA.	C57BL/6J.	Tet-inducible OE of human *LRRK2* WT with CaMKII promoter (neuron specific).	Not assessed.	No changes in beam breaks and latency to fall up to 6 months (Lin).
18	Wang L. et al., 2008; Lin et al., 2009	Human *LRRK2* G2019S – HA.	C57BL/6J.	Tet-inducible OE of human *LRRK2* G2019S with CaMKII promoter (neuron specific).	Not assessed in SNpc (limited expression). No neuronal loss in striatum or cortex up to 20 months (Lin).	Increased beam breaks at 12 and 18 months. Rearings normal up to 18 months (Lin).
19	Lin et al., 2009	Human *LRRK2* kinase domain deletion – HA.	C57BL/6J.	Tet-inducible OE of human *LRRK2* kinase domain deletion with CaMKII promoter (neuron specific).	Not assessed.	Not assessed.

**TABLE 4:** Transgenic OE of *LRRK2* using BAC in mouse models.

Transgenic overexpression BAC mouse models	Author(s), year(s)	Model (species, gene, WT or mutant, tag).	Background (strain).	Type of expression system.	DA neuronal loss.	Locomotor/behavioral changes.
1	Beccano-Kelly et al., 2015; Volta et al., 2015	Human *LRRK2* WT.	C57BL/6J [backcrossed from Melrose et al. (2010) by Beccano-Kelly].	OE of human *LRRK2* WT using BAC (RP-11 568G5).	Not assessed.	Decreased total distance, ambulatory time and rearings at 3–6 months (Beccano-Kelly). Decreased rearings at 12 months (Volta).
2	Volta et al., 2015	Human *LRRK2* G2019S.	C57BL/6J [backcrossed from Melrose et al. (2010)].	OE of human *LRRK2* G2019S using BAC (RP-11 568G5).	Not assessed.	Increased cylinder rearings at 6 months, but normal at 12 months.
3	Dächsel et al., 2010; Melrose et al., 2010	Human *LRRK2* WT.	FVB/N (backcrossed – Melrose).	OE of human *LRRK2* WT using BAC (RP-11 568G5).	No loss in SN up to 22–24 months (Melrose).	No changes in open field, beam-crossing test, gait footprint analysis inked footprint analysis or negative geotaxis test at 7–8 months (Melrose).
4	Dächsel et al., 2010; Melrose et al., 2010	Human *LRRK2* G2019S.	FVB/N (backcrossed).	OE of human *LRRK2* G2019S using BAC (RP-11 568G5).	No loss in SN up to 22–24 months (Melrose).	Increased mean path length and thigmotaxis (wall hugging) in open field at 7–8 months. No other changes in beam-crossing, gait footprint analysis or negative geotaxis tests (Melrose).
5	Dächsel et al., 2010	Human *LRRK2* Y1699C.	FVB/N (backcrossed).	OE of human *LRRK2* Y1699C using BAC (RP-11 568G5).	Not assessed.	Not assessed.
6	Li et al., 2007, 2010	Mouse *LRRK2* WT – FLAG.	C57BL/6J.	OE of mouse *LRRK2* WT using BAC (RP23-312I9).	No loss in SNpc at 12 months (2010).	Increased rearings at 6 and 12 months, total distance at 12 months and total move time at 12 months. Decreased slips/step at 12 months (2010).
7	Li et al., 2010	Mouse *LRRK2* G2019S – FLAG.	C57BL/6J.	OE of mouse *LRRK2* G2019S with mouse BAC (RP23-312I9).	No loss in SNpc at 12 months.	No changes in open field test, beam test, and gait stride test at 12 months.
8	Li et al., 2009	Human *LRRK2* WT.	FVB/NJ.	OE of human *LRRK2* WT with BAC.	No loss up to 9–10 months.	No changes in open field or cylinder test.
9	Li et al., 2009; Bichler et al., 2013; Dranka et al., 2013	Human *LRRK2* R1441G.	FVB/NJ.	OE of human *LRRK2* R1441G with human BAC (RP135).	No loss up to 9–10 months (Li).	Decreased rearings at 6 and 10–12 months (Li). Decreased rearings (cylinder), center activity (open field), and horizontal activity (open field) at 20 months (Bichler) (See Bichler for other important negative data). Decreased latency to fall and increased time on pole test at 16 months (Dranka).

**TABLE 5:** Rat transgenic *LRRK2* models.

	Author(s), year(s)	Model (species, gene, mutation, tag)	Background (strain)	Type of expression system	DA neuronal loss	Locomotor/behavioral changes
**Transgenic overexpression cDNA rat models**						
1	Zhou et al., 2009, 2011	Human *LRRK2* WT – HA.	Sprague-Dawley.	Tet-inducible OE of human *LRRK2* WT with a ubiquitous promoter (TRE-miniCMV).	Not assessed.	Not assessed.
2	Zhou et al., 2011	Human *LRRK2* G2019S – HA.	Sprague-Dawley.	Tet-inducible OE of human *LRRK2* G2019S with a ubiquitous promoter (TRE-miniCMV).	No loss in SNpc and LC at 18 months (compared to “tet-only” instead of non-transgenic).	Increased total distance (open field) at 18 months using temporary expression model (given doxycycline until 5 months).
**Transgenic overexpression BAC rat models**						
1	Sloan et al., 2016	Human *LRRK2* WT – YPet.	Sprague-Dawley.	OE of human *LRRK2* WT with BAC.	Not assessed.	No changes in rotarod, T-maze, or grip strength test.
2	Sloan et al., 2016	Human *LRRK2* G2019S – YPet.	Sprague-Dawley.	OE of human *LRRK2* G2019S with BAC.	No loss in SN at 18–21 months.	Decreased latency to fall at 3–6 and 18–21 months. Decreased alternations in T-maze at 18–21 months.
3	Sloan et al., 2016	Human *LRRK2* R1441C – Ypet.	Sprague-Dawley.	OE of human *LRRK2* R1441C with BAC.	No loss in SN at 18–21 months.	Decreased latency to fall, decreased alternations (T-maze) and increased gait disturbances at 18–21 months.
4	(Shaikh et al., 2015) (Taconic – Dr. Chenjian Li)	Human *LRRK2* R1441G.	Sprague-Dawley.	OE of human *LRRK2* R1441G under BAC.	No loss in SN or striatum up to 9 months.	No changes in forelimb placing test, adjusting steps test, footprint analysis, open field test, acoustic startle response and Morris water maze.
5	(Walker et al., 2014; West et al., 2014; Daher et al., 2015; Lee et al., 2015; Volpicelli-Daley et al., 2016) (Taconic – Dr. Chenjian Li)	Human *LRRK2* G2019S.	Sprague-Dawley.	OE of human LRRK2 G2019S under BAC (MJ Farrer Lab).	No changes (Daher).	Decreased latency to fall on rotarod at 6 months. No difference in cylinder and beam walking test (Walker). Postural instability at 8 months but was recovered at 12 months. Rearings increased at 12 months (Lee).

An emerging theme in transgenic mouse models is that overexpression of pathogenic *LRRK2* mutants, such as G2019S or R1441C, can induce PD-like phenotypes. Reported phenotypes include DA neuronal loss, disruption of dopamine homeostasis, ultrastructural changes, L-DOPA responsive locomotor defects, and pathological accumulations of tau and α-synuclein (Ramonet et al., 2011; Chen et al., 2012; Weng et al., 2016; Xiong et al., 2018). Several of these phenotypes have also been reproduced in rats, though without any obvious DA neuronal loss (Zhou et al., 2011; West et al., 2014; Sloan et al., 2016). In mice, DA neuron degeneration is typically observed at mid- to older age, occurring typically at 15–20 months in the SNpc (Ramonet et al., 2011; Weng et al., 2016; Xiong et al., 2018). Prior to cell death, DA neurons are frequently observed to exhibit abnormal morphology and reduced synaptic vesicles (Burke and O’Malley, 2013; Tsika et al., 2014; Liu et al., 2015; Weng et al., 2016). Decreased striatal dopamine content, dopamine metabolites, and evoked dopamine release also frequently occur in conjunction with DA neuronal death (Ramonet et al., 2011; Zhou et al., 2011; Liu et al., 2015; Sloan et al., 2016; Lim et al., 2018; Xiong et al., 2018). Consequently, these rodents exhibit locomotor defects, which are partially rescued with L-DOPA (Sloan et al., 2016; Weng et al., 2016; Xiong et al., 2018). To date, two studies using cDNA *LRRK2*-G2019S overexpression have been able to show both DA neurodegeneration and pathological inclusions (Chen et al., 2012; Xiong et al., 2018). In one such model, phosphorylated tau was increased at 12 months in the SNpc, in parallel with DA neuronal loss (Chen et al., 2012). The other reported an increase in phosphorylated and high molecular weight α-synuclein in the striatum and ventral medial body at 24 months of age (Xiong et al., 2018).

Although transgenic OE rodent models can capture many of the cardinal features of PD patients to various extents, there are several key caveats to bear in mind. For example, the question of whether DA neuronal loss occurs in a cell-autonomous or non-cell-autonomous manner has arisen, due to cell type-specific expression. Only mouse models which employ a DA neuron specific (TH) or neuronal transgene (CMV enhancer/PDGF-β) promoter has been able to induce DA neurodegeneration (Ramonet et al., 2011; Chen et al., 2012; Weng et al., 2016; Xiong et al., 2018). Of note, other neuronal transgene promoters, such as Thy1.2 and CaMKII, have failed to induce DA neuronal death, possibly owing to a lack of sufficient expression in midbrain DA neurons (Tsika et al., 2014; Garcia-Miralles et al., 2015). Alternative rodent models generated using a BAC approach, which use endogenous *LRRK2* promoter to drive the expression has presented with more subtle phenotypes, such as changes in dopamine homeostasis and mild behavioral or locomotor dysfunction (Tables 4, 5).

One clear advantage of rodent OE models is that they have enabled the study of neurodegenerative mechanisms. One potential mechanism of action is LRRK2-mediated phosphorylation of synaptic proteins with known functions in vesicle endocytosis. Specifically, LRRK2 has been shown to phosphorylate the synaptic proteins synaptojanin 1 (*SYNJ1*), Endophilin A1 (*SH3GL2*) and auxilin (*DNAJC6*) (Matta et al., 2012; Arranz et al., 2015; Pan et al., 2017; Nguyen and Krainc, 2018). Broadly speaking, these studies have proposed that increased LRRK2-kinase activity disrupts the physiological function of these presynaptic trafficking proteins, resulting in defects in synaptic vesicle endocytosis (Pan et al., 2019).

### Mouse *LRRK2* KI Models

Currently, the disease mutations of *LRRK2* knock-in (KI) models have only been developed in mice (Table 6). Unlike OE models, KI models do not suffer from potential overexpression artifacts or interspecies differences. However, *LRRK2* KI mouse models have failed to show DA neuron degeneration or α-synuclein pathology. Rather, these models exhibit neurophysiological changes, altered DA homeostasis, and modest behavioral abnormalities.

**TABLE 6:** Mouse *LRRK2* KI models.

Mouse *LRRK2* KI models	Author(s), year(s)	Model (species, gene, mutation, tag)	Background (strain)	DA neuronal loss	Locomotor/behavioral changes	Other notes
1	Giesert et al., 2017	Mouse *LRRK2* R1441C KI.	C57BL/6J (backcrossed).	No loss in SN up to 28 months.	Increased time on pole test, increased total slips (beam test), and increased time on ladder test at >24 months. Gait analysis shows decreased front paw angle on CatWalk at 26 months. Reduction in odor sensitivity and discrimination at 24–26 months. Decrease in time spent swimming on forced swim test at 8–15 months. Tail suspension test altered at 8 months in females (See Giesert for important negative data).	No synuclein or tau pathology. R1441C KI line has locomotor or behavioral symptoms that may indicate prodromal/early phase of PD in humans.
2	(Steger et al., 2016) (Eli Lilly)	Mouse *LRRK2* G2019S KI.	C57BL/6J.	Not assessed.	Not assessed.	
3	(Steger et al., 2016) (Michael J. Fox Foundation)	Mouse *LRRK2* A2016T KI (kinase inhibitor resistant).	C57BL/6NJ.	Not assessed.	Not assessed.	
4	Ito et al., 2016	Mouse *LRRK2* Kinase Dead (D2017A) KI.	C57BL/6J (backcrossed).	Not assessed.	Not assessed.	
5	Ito et al., 2016; Zhou et al., 2018	Mouse *LRRK2* [S910A + S935A] KI.	C57BL/6 (backcrossed).	Not assessed.	Increased latency to fall at 40 rpm but not 50 rpm on rotarod (Zhou).	Reduced astrocytes in dorsolateral striatum at 18 months. Increased α-synuclein in dorsolateral striatum at 3 months.
6	(Matikainen-Ankney et al., 2016, 2018) (Eli Lilly)	Mouse *LRRK2* G2019S KI.	C57BL/6NTac.	Not assessed.	More resistant to chronic social defeat stress (CSDS). Altered self-care based on grooming time. No difference on rotarod or elevated plus maze test (2018).	Increased sEPSC frequency in dorsal striatal SPNs at P21 (restored with kinase inhibition). SPNs at P21 had greater spine head width (2016). Decreased sEPSC amplitude in NAc SPNs. Mice lack CP-AMPAR at baseline and post-CSDS. Unable to form LTP in dorsal striatal SPNs (2018).
7	(Matikainen-Ankney et al., 2016) (Eli Lilly)	Mouse *LRRK2* Kinase Dead (D2017A) KI.	C57BL/6NTac.	Not assessed.	Not assessed.	
8	Liu et al., 2014	Mouse *LRRK2* R1441G KI.	C57BL6/N (backcrossed).	No loss in SNpc up to 18–22 months. No TH cell loss in striatum at 18–22 months.	No changes at 3 and 12 months in open field.	No synuclein, tau or ubiquitin pathology. Alterations in open field and DA uptake in response to reserpine (depletes DA in striatum).
9	Dächsel et al., 2010; Beccano-Kelly et al., 2014; Yue et al., 2015; Volta et al., 2017	Mouse *LRRK2* G2019S KI.	C57BL/6.	No loss in SN up to 18–20 months (Yue).	Increased distance traveled and latency to fall at 6 months (not seen at 12 months) (Yue). Increased rearing at 6 months (not seen at 12 months) (Volta).	Increased phospho-tau in corpus callosum and midbrain at 18 months. Decrease in extracellular DA at 12 months. Dose dependent increase in kinase activity. Decrease fission/fusion of mitochondria at 15 months (Yue). Increased sEPSC frequency in DIV21 cortical cultures. Reduced synapsin 1 phosphorylatio in DIV21 cortical cultures (Beccano-Kelly). Increased sEPSC frequency in striatal SPNs at 1–3 months and increased dopamine transmission (Volta).
10	Herzig et al., 2011; Longo et al., 2014	Mouse *LRRK2* G2019S KI.	C57BL/6J or BALB/c.	No loss in striatum at 20 and 22 months (Herzig).	Decreased immobility time (bar time) at 6–19 months. Increased number of steps (drag test) at 6 –19 months. Decreased immobility time (open field) at 15 months. Increased total distance traveled (open field) at 15 months (Longo).	No synuclein pathology. Increased diastolic blood pressure (Herzig).
11	Herzig et al., 2011; Longo et al., 2014	Mouse *LRRK2* Kinase Dead (D1994S) KI.	C57BL/6 or BALB/c.	Not assessed.	No changes in bar test, drag test, rotarod and open field up to 15 months (Longo).	Increased kidney weight at 6 months. Darkened kidney (Herzig).
12	Tong et al., 2009; Nichols et al., 2010; Parisiadou et al., 2014	Mouse *LRRK2* R1441C KI.	C57BL/6.	No loss in SNpc or LC at 3, 12, and 22 months (Tong).	No changes in open field, rotarod and acoustic startle response. No change in AMPH injection compared (Tong).	Decreased sensitivity for firing by DA (Tong). Excess PKA activity in SPNs (Parisiadou).

Two independent groups have reported that the *LRRK2*-G2019S KI mice exhibit increased frequency of spontaneous excitatory post-synaptic potentials (sEPSCs) in spiny projection neurons (SPNs) of the striatum (Matikainen-Ankney et al., 2016; Volta et al., 2017). Young (1–3 months) G2019S KI mice also exhibit elevated dopamine release upon repeated stimulation (Volta et al., 2017). These changes are mirrored by increases in total distance moved and rearings indicative of hyperkinesia at a young age (Longo et al., 2014; Yue et al., 2015; Volta et al., 2017). However, in older mice (>12 months), extracellular levels of dopamine appear decreased and hyperkinetic behavior is reduced (Yue et al., 2015; Volta et al., 2017). Perhaps surprisingly, a recent study has reported that young G2019S KI mice are more resilient to chronic social defeat stress (CSDS) (Matikainen-Ankney et al., 2018). The authors suggest that this may be due to the inability for calcium-permeable AMPA receptors (CP-AMPARs) to integrate into the synaptic membrane, blocking the formation of long-term potentiation (LTP). However, whether the synaptic changes at young adulthood in rodents confer altered non-motor or motor phenotypes in late-onset PD remains unclear.

## *Drosophila LRRK2* Models

*Drosophila LRRK2* models can offer several advantages over rodents. Firstly, the relatively short lifespan of *Drosophila* (∼2 to 3 months) allows age-dependent changes in phenotypic variability to manifest quicker than in rodent models (e.g., synuclein pathology; Feany and Bender, 2000). In addition, the presence of the *UAS-GAL4* system in several *Drosophila* lines can create a diverse range of genetic manipulations. Finally, the dopaminergic system in *Drosophila* consists of six well-defined neuronal clusters that can easily be quantified to assay DA-specific neuronal death.

### *Drosophila dLRRK* KO Models

Unlike rodents and humans, which possess both *LRRK1* and *LRRK2* genes, *Drosophila* only possesses a single *LRRK2* ortholog, *dLRRK*. dLRRK is 2,445 amino acids in length and shares an overall sequence identity of 24% with human LRRK2 (Wang D. et al., 2008). Like *LRRK2* KO rodent models, loss of *dLRRK* does not cause DA neurodegeneration (Table 7) (Imai et al., 2008; Wang D. et al., 2008; Tain et al., 2009). Several studies have, however, reported locomotor deficits and loss of fertility in *dLRRK* KOs (Lee et al., 2007; Imai et al., 2008; Tain et al., 2009). An unclear point of investigation is whether *dLRRK* KOs are sensitive or protective to treatment with reactive oxygen species (ROS) with two studies observing conflicting evidence for H_2_O_2_ on mortality (Imai et al., 2008; Wang D. et al., 2008). Future studies should evaluate reasons for this discrepancy and probe potential mechanisms for ROS sensitivity or protection in *dLRRK* KOs.

**TABLE 7:** *Drosophila dLRRK* KO models.

	Author(s), year(s)	Model	DA neuronal loss	Locomotor/behavioral changes
***Drosophila dLRRK* KO Models**				
1	Dodson et al., 2014	Partial loss by early stop codon [*dLRRK* (+/−)].	No change in PPM1/2, PPM3, PPL1, PPL2, VUM, and PAL clusters.	Not assessed.
2	Lee et al., 2007; Imai et al., 2008; Wang D. et al., 2008; Tain et al., 2009	KO of *dLRRK* by piggy-BAG insertion [*dLRRK* (−/−)].	Loss of TH staining is decreased in DM and PM clusters at 3 and 10 days (Lee). No loss in PPM1/2, PPL1, PPM3 (Imai). No change in number or distribution (Wang). No change in PPL1 cluster (Tain).	Climbing deficits at 3 days and loss of fertility in females (Lee). Smaller abdomen and decreased fertility (Imai). No behavioral abnormality or decrease in fertility (Wang). Tendency for decreased climbing ability (Tain).
3	Lee et al., 2007	KO of *dLRRK* by deletion of 3′ UTR region and EP element [*dLRRK* (−/−)].	Not assessed.	Climbing deficits at 3 days and loss of fertility in females (data not shown).

### *Drosophila* Transgenic *LRRK2* Models

As with rodent OE models, *Drosophila* OE of human *LRRK2* G2019S and R1441C has resulted in DA neuronal loss, disruptions of dopamine homeostasis, and L-DOPA responsive locomotor defects (Table 8). Interestingly, other variants such as I1122V, Y1699C, I2020T, and G2385R have also been shown to cause DA neuronal loss in *Drosophila* (Imai et al., 2008; Liu et al., 2008; Ng et al., 2009; Venderova et al., 2009; Lin et al., 2010; Godena et al., 2014). This loss occurs at mid- to older age (35–60 days) and is commonly seen in the PPL1 DA neuronal cluster (Imai et al., 2008; Liu et al., 2008; Ng et al., 2009; Venderova et al., 2009). In addition to cell loss, transgenic models also exhibit decreased DA content and reduced climbing ability starting at birth or at 10 days, respectively (Imai et al., 2008; Ng et al., 2009). Several studies have also reported sensitivity to ROS in flies expressing pathogenic *LRRK2* mutations (Imai et al., 2008; Ng et al., 2009).

**TABLE 8:** *Drosophila* transgenic *LRRK2* models.

	Author(s), year(s)	Model (species, gene, mutation, tag)	DA neuronal loss	Locomotor/behavioral changes
**Transgenic overexpression *Drosophila* models**				
1	Lin et al., 2010	Human *LRRK2* WT – FLAG.	Not assessed.	Not assessed.
2	Lin et al., 2010	Human *LRRK2* G2019S – FLAG.	Loss at 4 weeks in dorsolateral and dorsomedial groups with *ddC* (dopa decarboxylase) promoter (dopamine and serotonin neuron specific).	Locomotor deficits (climbing index) at 3 weeks and decreased viability at 4 weeks.
3	Lin et al., 2010; Hindle et al., 2013	Human *LRRK2* G2385R – FLAG.	Not assessed.	Not assessed.
4	Lin et al., 2010; Hindle et al., 2013; Godena et al., 2014	Human *LRRK2* R1441C – FLAG	Not assessed.	Not assessed.
5	Lin et al., 2010; Hindle et al., 2013	Human *LRRK2* G2019S-K1906M- FLAG.	Not assessed.	Not assessed.
6	Venderova et al., 2009	Human *LRRK2* WT.	Loss at 50 days with TH promoter (catecholaminergic cell specific) in PPL1 and PPM1/2 neuronal clusters.	Decreased locomotion starting at 10 days (time required to recover from tapping).
7	Venderova et al., 2009; Hindle et al., 2013	Human *LRRK2* I1122V.	Loss at 50 days with TH promoter in PPL1 and PPM1/2 neuronal clusters.	Decreased locomotion starting at 10 days (time required to recover from tapping).
8	Venderova et al., 2009	Human *LRRK2* Y1699C.	Loss at 50 days with TH promoter in PPL1 and PPM1/2 neuronal clusters.	Decreased locomotion starting at 10 days (time required to recover from tapping).
9	Venderova et al., 2009; Hindle et al., 2013	Human *LRRK2* I2020T.	Loss at 50 days with TH promoter in PPL1 and PPM1/2 neuronal clusters (most dramatic compared to others).	Decreased locomotion starting at 10 days (time required to recover from tapping).
10	Ng et al., 2009	Human *LRRK2* WT – Myc	No loss.	No climbing deficits.
11	Ng et al., 2009	Human *LRRK2* G2019S – Myc.	Loss at 60 days old under *ddC* promoter.	Climbing is impaired at 60 days with *ddC* promoter.
12	Ng et al., 2009	Human *LRRK2* Y1699C – Myc.	Loss at 60 days old under *ddC* promoter.	Climbing is impaired at 60 days with *ddC* promoter.
13	Ng et al., 2009	Human *LRRK2* G2385R – Myc.	Loss at 60 days old under *ddC* promoter.	No climbing deficits.
14	Liu et al., 2008; Hindle et al., 2013; Godena et al., 2014	Human *LRRK2* WT – FLAG.	Loss at 35 days using *ddc* but 49 days with *elav* promoter (pan-neuronal) (Liu).	Climbing impaired at 4 weeks with *ddC* promoter and 6 weeks with *elav.*
15	Liu et al., 2008; Hindle et al., 2013; Godena et al., 2014	Human *LRRK2* G2019S – FLAG.	Loss at 35 days using *ddC* promoter and 35 days with *elav* promoter (Liu).	Climbing impaired at 4 weeks with *ddC* promoter and 6 weeks with *elav* (more severe than human *LRRK2* WT OE model) (Liu).
16	Imai et al., 2008; Lee et al., 2010; Hindle et al., 2013	*dLRRK* WT.	No loss in PPM1/2, PPL1, and PPM3 (Imai)	Not assessed
17	Imai et al., 2008; Hindle et al., 2013; Godena et al., 2014	*dLRRK* Y1383C (corresponding to human Y1699C).	Loss at 60 days old using *ddC* and *elav* promoter in PPM1/2 and PPL1 (Imai).	Decreased fertility in females (Imai).
18	Imai et al., 2008; Lee et al., 2010; Hindle et al., 2013	*dLRRK* I1915T (corresponding to human I2020T).	Loss at 60 days old using *ddC* and *elav4* promoter in PPM1/2 and PPL1 (Imai).	Decreased fertility in females and climbing deficits in 45-day old flies (Imai).
19	Imai et al., 2008; Lee et al., 2010; Hindle et al., 2013	*dLRRK* 3KD (K1781, D1882A, and D1912A) “Triple Kinase Dead”.	No loss in PPM1/2, PPL1, and PPM3 (Imai).	No climbing deficits (Imai).
20	Lee et al., 2007	*dLRRK* WT – FLAG.	No loss.	No deficits.
21	Lee et al., 2007; Godena et al., 2014	*dLRRK* R1069C (corresponding to R1441C).	No loss (Lee).	No deficits.

Unlike rodent models, OE of human WT *LRRK2* has also been reported to induce DA neurodegeneration (Liu et al., 2008). In addition, OE of *Drosophila* WT *dLRRK* (under a TH promoter) has also been shown to cause neurodegeneration (Imai et al., 2008). These models raise several questions, including whether DA neuronal loss may be caused by expression of a different species’ *LRRK2* and whether cell-type specific expression can cause DA neuronal loss.

## *Caenorhabditis elegans LRRK2* Models

*Caenorhabditis elegans* have a well-characterized CNS with a total of 302 neurons, 8 of which are dopaminergic. Unlike rodents, the simplicity of the *C. elegans’*s nervous system has allowed for quantification and visualization of DA neuron morphology *in vivo* (Yao et al., 2010). In addition, its shorter lifespan, small size and cost-effectiveness have facilitated high-throughput drug screening (Maulik et al., 2017). Like *Drosophila*, *C. elegans* has one single ortholog of *LRRK2*, *Lrk-1*, which is broadly expressed in head and tail neurons, the hypodermis, intestine, and muscles (Sakaguchi-Nakashima et al., 2007). Both *Lrk-1* KO and transgenic OE models of *LRRK2* have been reported in *C. elegans*.

### *C. elegans Lrk-1* KO Models

Like other *LRRK2* KO models, loss of *Lrk-1* does not lead to the degeneration of DA neurons or striking locomotor phenotypes (Sakaguchi-Nakashima et al., 2007; Saha et al., 2009; Sämann et al., 2009; Yao et al., 2010). *C. elegans* do, however, demonstrate a mis-localization of synaptic vesicles in DA neurons *in vivo* (Sakaguchi-Nakashima et al., 2007; Sämann et al., 2009). In addition, *Lrk-1* KOs have been found to be more sensitive to ER stressors, such as tunicamycin, which has been recently observed to induce the recruitment of LRRK2 from the *trans*-Golgi to the lysosome in a Rab29-dependent manner (Sämann et al., 2009; Kuwahara et al., 2016; Eguchi et al., 2018). Both of these processes depend on the phosphorylation of Rab GTPases by LRRK2, and future studies in *C. elegans* may reveal more mechanistic insight *in vivo*.

### *C. elegans LRRK2* Transgenic Models

The OE of human *LRRK2* G2019S and R1441C in *C. elegans* has been shown to cause DA neurodegeneration, reduced dopamine levels, and locomotor dysfunction (Sämann et al., 2009; Yao et al., 2010; Liu et al., 2011). Interestingly, two conflicting studies suggest that LRRK2-mediated neurodegeneration may depend on either GTPase or kinase activity. One study suggests that OE of human *LRRK2* K1347A (a defective GTP binding mutant) does not produce Parkinsonian phenotypes in *C. Elegans*, inferring that GTPase activity is essential for toxicity (Yao et al., 2010), while another indicates that the kinase dead human *LRRK2* OE also does not produce loss of DA neurons (Liu et al., 2011). Furthermore, OE of human *LRRK2* WT has also been shown to cause DA neurodegeneration (Yao et al., 2010). Future *C. elegans* transgenic OE models will have to clarify these discrepancies and use better GTPase inactive mutants in *C. elegans* to assess for toxicity.

## Zebrafish *LRRK2* Models

Zebrafish (*Danio rerio*) is an attractive model due to its well-characterized neuronal circuitry, conserved neurobiochemical mechanisms between humans, optical transparency, small size, and ease of drug administration (Vaz et al., 2018). The lifespan of zebrafish is relatively long compared to other rodent models, with 71% survivability at 5 years, making it ideal for aging research (Vaz et al., 2018). Like *Drosophila* and *C. elegans*, zebrafish possess a sole ortholog to human *LRRK2*, called *zLRRK2*, sharing a 47% amino acid sequence identity with humans (Sheng et al., 2010).

### Zebrafish *zLRRK2* KD Models

Since an initial study reported that *zLRRK2* KO is embryonically lethal, a targeted knock-down (KD) approach has been used as an alternative to reduce the expression of *zLRRK2* (Sheng et al., 2010). This initial study saw that targeting the WD40 domain of *zLRRK2* using morpholino oligonucleotides resulted in the loss of diencephalon DA neurons and locomotor defects (Sheng et al., 2010). A later study supported this finding and also observed α-synuclein aggregates in multiple brain regions (Prabhudesai et al., 2016). However, one study using a dosage of morpholinos in between these two studies achieved a greater KD of *zLRRK2*, but could not replicate these phenotypes (Ren et al., 2011). These studies may show conflicting results due to the reported off-target effects of morpholinos, and thus different approaches should be considered (Kok et al., 2015). As such, further study is required to establish a consistent theme in zebrafish *zLRRK2* KD models.

### Zebrafish *LRRK2* Mutant Models

Limited zebrafish mutant models have been developed, but a recent report using zinc finger nucleases (ZFNs) was able to introduce a mutation in the WD40 domain of *zLRRK2* to generate a KI model (Sheng et al., 2018). They observed increases in locomotion in the adult stage and a weakened antibacterial response. Another report using transient overexpression of human *LRRK2* WT and G2019S in zebrafish embryos was able to see disruptions in the ubiquitin-proteasome system (UPS) (Lichtenberg et al., 2011). Future studies will have to develop more mutant models of zebrafish models in order to gain more phenotypic insights.

## Concluding Remarks and Future Perspectives

Here we highlight several phenotypic themes found in *LRRK2*-associated PD animal models, with an emphasis on rodents.

*LRRK2* KO models in mice, rats, *Drosophila*, and *C. elegans* have not produced DA neurodegeneration. Rather, phenotypic changes have been observed primarily in peripheral tissues such as the kidneys and lungs. Interestingly, primates treated with *LRRK2* kinase inhibitors have also show disruptions in the kidneys and lungs with no obvious neuropathological changes (Fuji et al., 2015). Future models should work to uncover other novel phenotypes associated with *LRRK2* KO models, which can be indicative of LRRK2 kinase inhibitor treatment, thereby facilitating the investigation of drug-based toxicity or efficacy. By contrast, transgenic OE of pathogenic *LRRK2* in mice and *Drosophila* has been shown to cause robust DA neurodegeneration, tau and α-synuclein pathology, locomotor/behavioral deficits, alterations in DA homeostasis, and L-DOPA-responsive behavior. A recently reported mouse model was found to have many of these features including DA neurodegeneration, locomotor changes and α-synuclein pathology, and it may be useful for determining drug efficacy (Xiong et al., 2018). Other phenotypes, such as tau pathology and cognitive, behavioral, and peripheral organ changes have not been assessed in this model and could provide other important clinical measures. Finally, *LRRK2* KI models used in mice and zebrafish have produced neurophysiological changes and modest behavioral or locomotor abnormalities. These phenotypes seen can be indicative of early or prodromal PD, and with careful behavioral analysis, such as one done by Giesert et al. (2017) in a R1441C KI mouse model, it can reveal other important phenotypes.

An emerging area in LRRK2 biology that may help guide future *LRRK2* models is the role of Rab proteins and whether they can be used as an *in vivo* biomarker for LRRK2 activity. LRRK2 is capable of phosphorylating Rab3A/B/C/D, Rab8A/B, Rab10, Rab12, Rab29, Rab35, and Rab43 (Steger et al., 2016, 2017). Using *LRRK2* G2019S, R1441C, kinase-dead, and phosphomimetic (S910A + S935A) KI rodent models, the endogenous phosphorylation of Rab proteins can be carefully evaluated in a tissue-specific manner. This should provide important clues for cell-type and region-specific readouts of LRRK2 activity (Ito et al., 2016; Lis et al., 2018). Furthermore, a recent study has highlighted LRRK2’s role at the lysosome, where LRRK2 is recruited onto stressed lysosomes by Rab29 and phosphorylates Rab8A and Rab10 (Eguchi et al., 2018). Interestingly, Rab8A KO mice have been developed, which may give important insights into LRRK2 biology at the lysosome (Sato et al., 2007). Furthermore, *RAB29* was also observed to be a risk factor for the development of PD, and future studies should investigate a potential unifying mechanism for LRRK2 and Rab29 in PD pathogenesis (MacLeod et al., 2013; Beilina et al., 2014; Nalls et al., 2019).

Another emerging area in *LRRK2* animal models is the role of LRRK2 in producing immune system abnormalities. A recent *LRRK2* phosphomimetic (S910A + S935A) KI mouse model has shown a reduction in astrogliosis, while a transgenic OE of LRRK2-G2019S in mice exhibited an increase in astrogliosis (Xiong et al., 2018; Zhou et al., 2018). Another study using G2019S KI mice has exhibited astrogliosis using a sub-toxic dose of MPTP dose to cause DA neurodegeneration (Arbez et al., 2019). Investigation of LRRK2 and astrogliosis using these models may help elucidate potential pathogenic mechanisms for LRRK2 in the CNS.

*LRRK2* animal models have thus facilitated our understanding of LRRK2 biology, have led us to determine PD pathogenic mechanisms, and have facilitated the discovery of novel phenotypes in LRRK2 pathogenesis in PD and therapeutic development.

## Author Contributions

SS, GH, and ZY wrote and edited the manuscript. DL had curated information on *C. elegans* and zebrafish models with assistance from BT. IC and MB provided topic specific knowledge on LRRK2 and guided sections in writing. All authors contributed to the article and approved the submitted version.

## Conflict of Interest

The authors declare that the research was conducted in the absence of any commercial or financial relationships that could be construed as a potential conflict of interest. The handling Editor HR declared a past co-authorship with the author ZY.
